# Supplementary material for: Quantitative global studies reveal differential translational control by start codon context across the fungal kingdom
Source: Nucleic Acids Res. 2020 Feb 5;48(5):2312–31. doi: 10.1093/nar/gkaa060 (PMC7049704; doi:10.1093/nar/gkaa060)
Supplement: gkaa060_Supplemental_Files [file gkaa060_supplemental_files.zip › Wallace_Crypto_AUG_suppdata_EW16Dec2019.pdf]

Quantitative global studies reveal differential translational control by start codon context across the fungal kingdom.

Edward Wallace, Corinne Maufrais, Jade Sales-Lee, Laura Tuck, Luciana de Oliveira, Frank Feuerbach, Frédérique Moyrand, Prashanthi Natarajan, Hiten D. Madhani, Guilhem Janbon.

Supplementary data

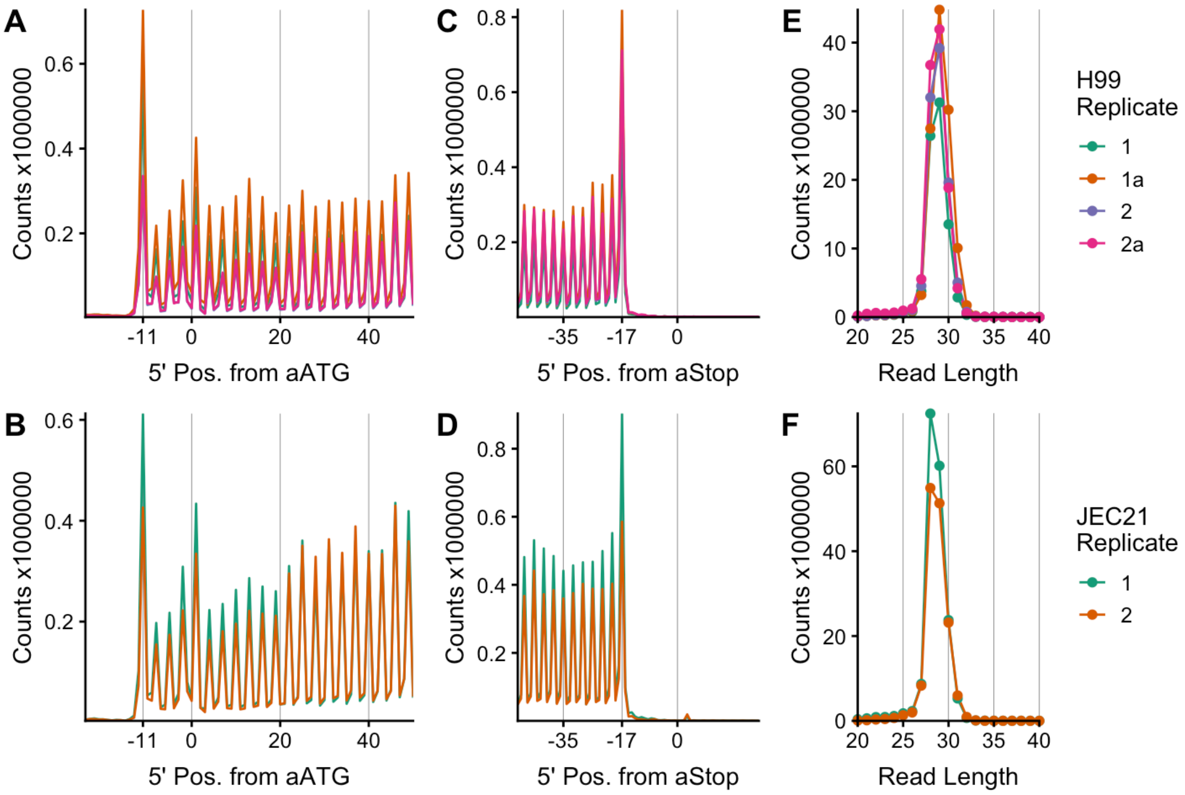

Figure S1: Ribosome profiling data passes quality control metrics. Metagene profiles of mapped 5' ends of ribosome-protected fragment counts at the 5' end (A,B) and 3' end (C,D) of ORFs, showing 3-nucleotide periodicity indicative of active translation starting at the annotated start codon and ending at the annotated stop codon. Ribosome protected fragment length is of a consistent length with other studies (E,F). Top row is data from 4 replicates of *C. neoformans* H99, bottom row form 2 replicates of *C. deneoformans* JEC21. These figures were made using RiboViz (43).

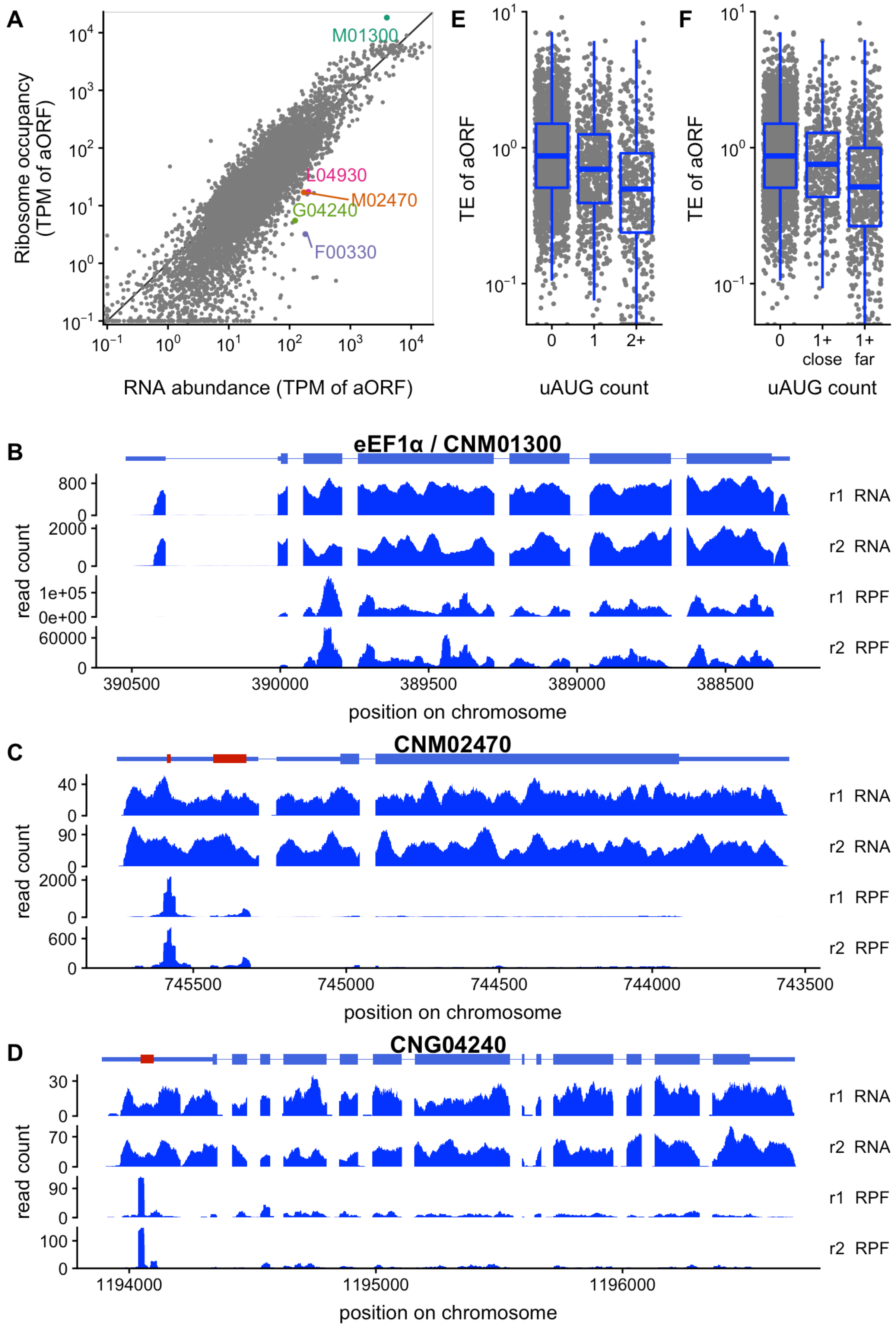
A
Ribosome occupancy (TPM of aORF)
RNA abundance (TPM of aORF)
M01300
L04930
M02470
G04240
F00330
E
TE of aORF
uAUG count
0
1
2+
F
TE of aORF
uAUG count
0
1+ close
1+ far
B
eEF1α / CNM01300
read count
r1 RNA
r2 RNA
r1 RPF
r2 RPF
position on chromosome
C
CNM02470
read count
r1 RNA
r2 RNA
r1 RPF
r2 RPF
position on chromosome
D
CNG04240
read count
r1 RNA
r2 RNA
r1 RPF
r2 RPF
position on chromosome

Figure S2: Upstream AUGs repress translation in *C. deneoformans*. A, translation regulation of annotated ORFs (aORFs) in *C. deneoformans* JEC21 growing exponentially in YPD at 30°C. B, Translation elongation factor eEF2/CNM01300 (CNAG_06125 homolog) has high ribosome occupancy in the annotated ORF. Translationally repressed mRNAs CNM02470 (CNAG_06246 homolog, C) and CNG04240 (CNAG_03140 homolog, D) have high ribosome occupancy in uORFs in the transcript leader (red), and low ribosome occupancy in the aORF. Only the first of 5 uORFs in CNG04240 is shown, and only transcript isoform t01 is shown, excluding the annotated TL intron in isoform t02. E, uAUGs are associated with lower translation efficiency (TE) of annotated ORFs. F, only uAUGs far from the transcription start site are associated with low TE. See figure 2 for full explanation.

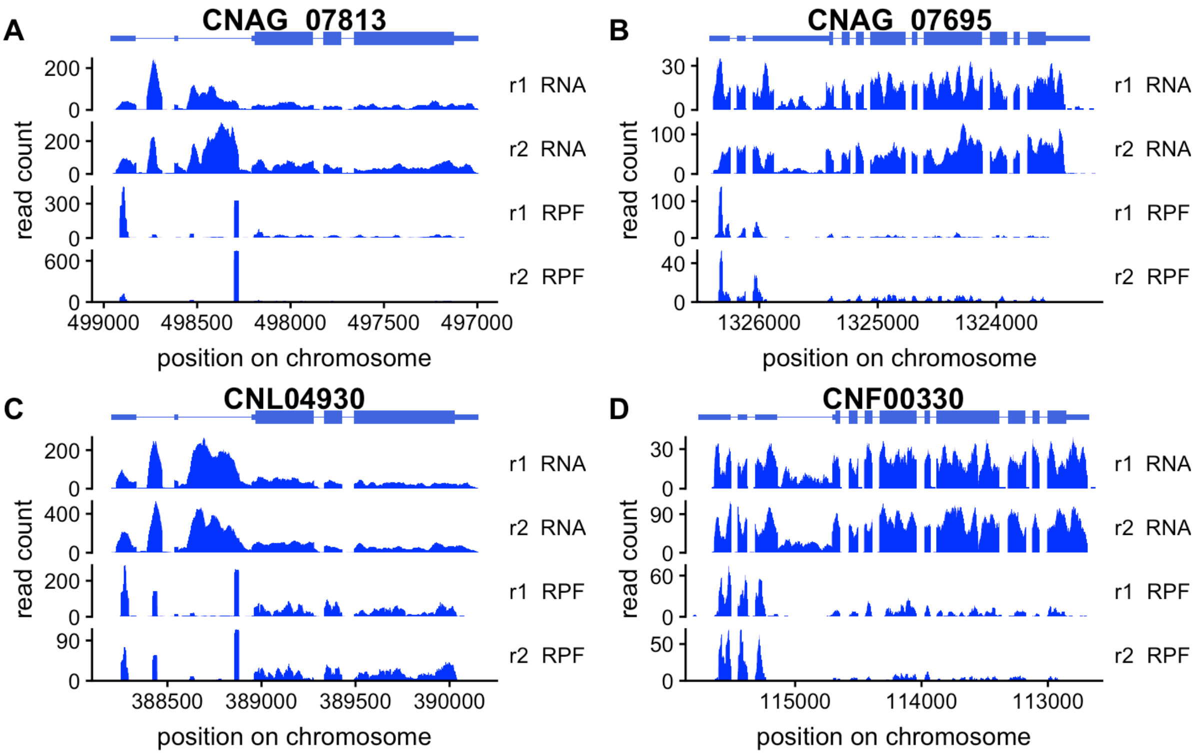

Figure S3: Further examples of upstream AUG and 5'-end regulation in *C. neoformans* and *C. deneoformans*. CNAG_07813 (A) and CNL04930 (C) are paralogs, and in addition to an upstream ORF with ribosome occupancy, they have an intronically encoded non-coding RNA in the TL. CNAG_07695 (B) and CNF00330 (D) are paralogs, and in addition to an upstream ORF with ribosome occupancy, they have an alternatively-spliced intron in the TL that is not occupied by ribosomes.

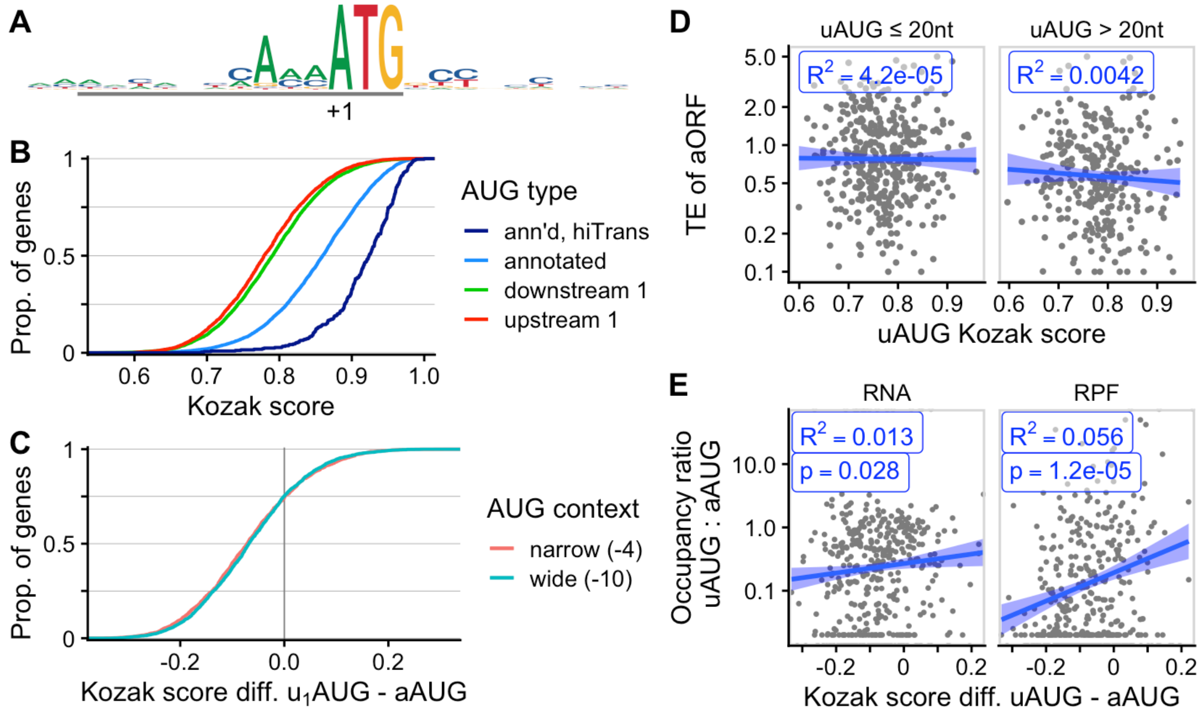

Figure S4: AUG sequence context is associated with translation in *C. deneoformans*. As for Fig 3, but with data from *C. deneoformans* JEC21.

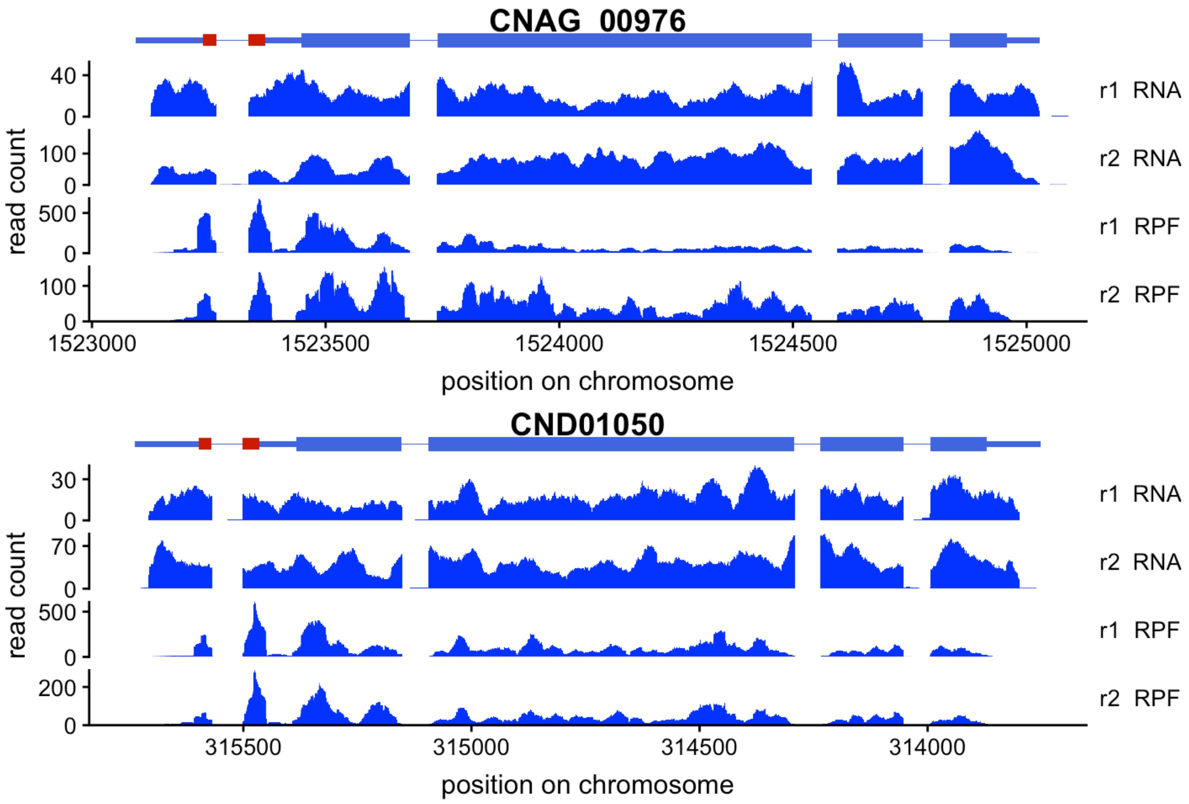

Figure S5: Carbamoyl-phosphate synthase CPA1 homologs have a conserved uORF that is occupied by ribosomes in *C. neoformans* (CNAG_00976, top) and *C. deneoformans* (CND01050, bottom).

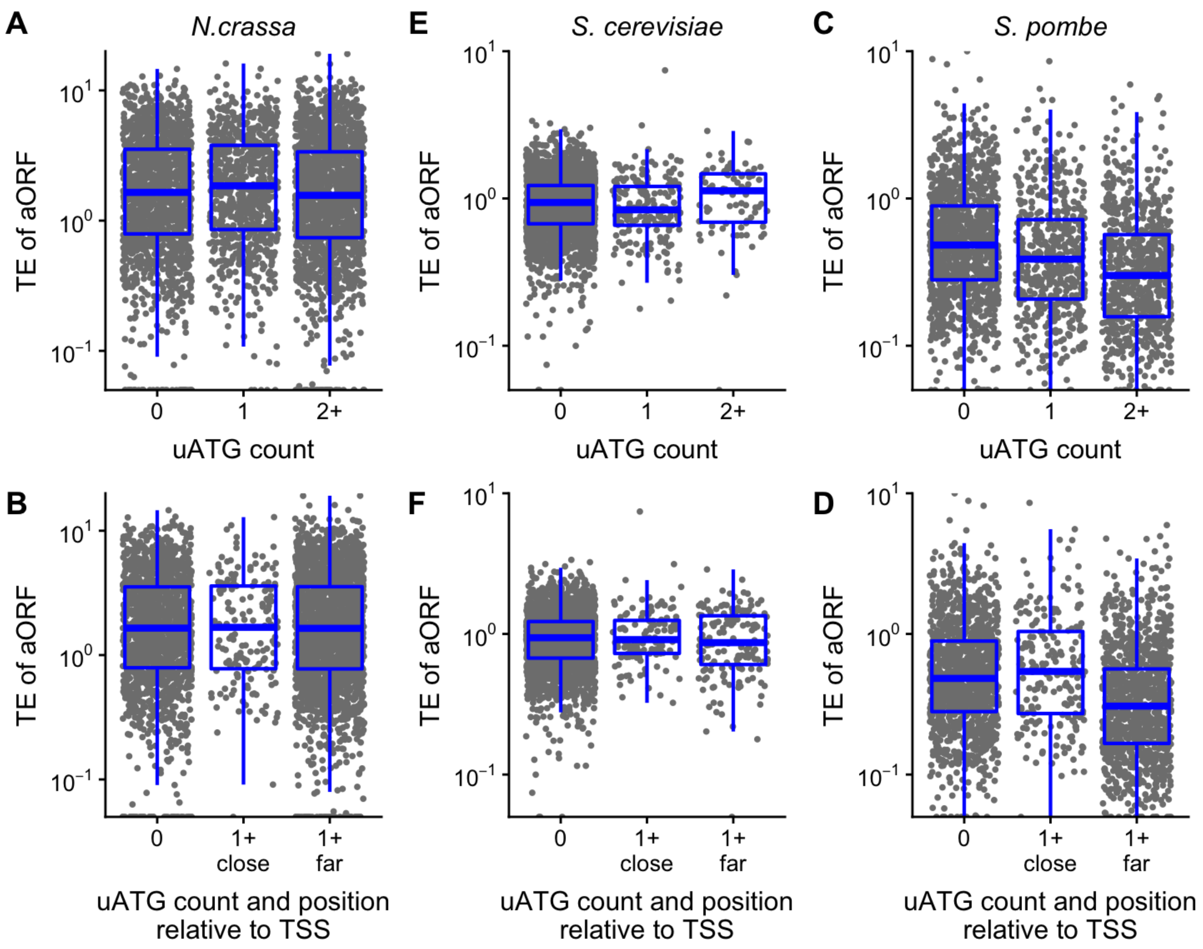

Figure S6: Effect of uATGs on translational efficiency in *N. crassa, S. pombe*, and *S. cerevisiae*.

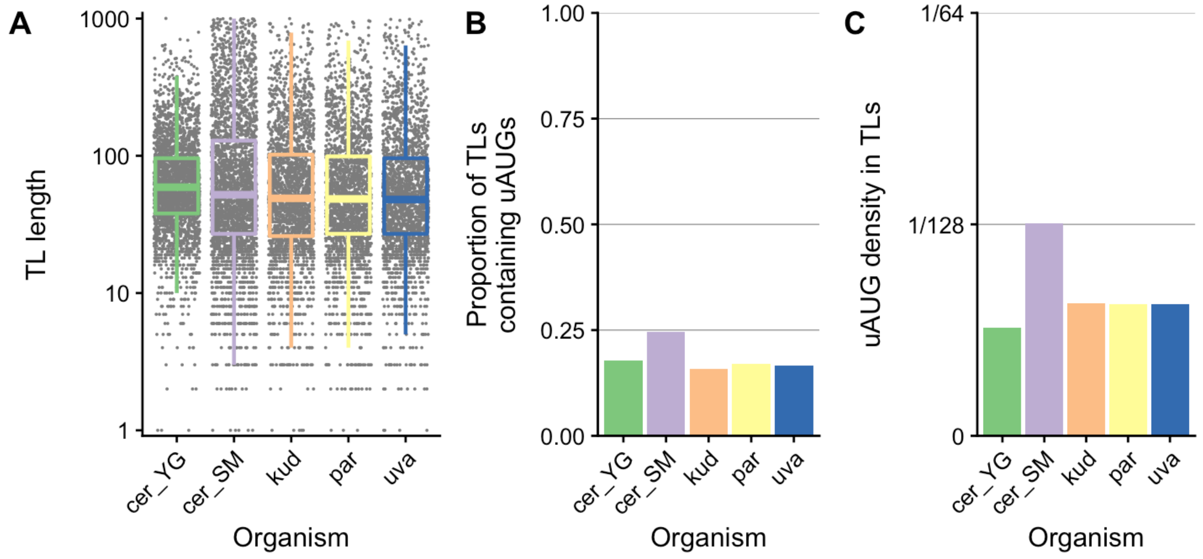

Figure S7: *Saccharomyces sensu stricto* species have short AUG-poor transcript leaders. We compared transcript leader length (A), proportion of transcript leaders containing uAUGs (B), and density of uAUGs in transcript leaders (C) between annotations of *Saccharomyces* yeasts. Annotations are abbreviated as: cer_YG, *S. cerevisiae* S288C from the *Saccharomyces* genome database (Cherry et al 2013); cer_SM, *S. cerevisiae* S288C from (Spealman et al. 2018); kud, *S. kudriavzevii* FM1340, par, *S. paradoxus* CBS432, uva, *S. bayanus* var. *uvarum* JRY9191, the latter 3 also from (Spealman et al. 2018). Note that all these annotations show a similar median leader length of 48-60 nucleotides.

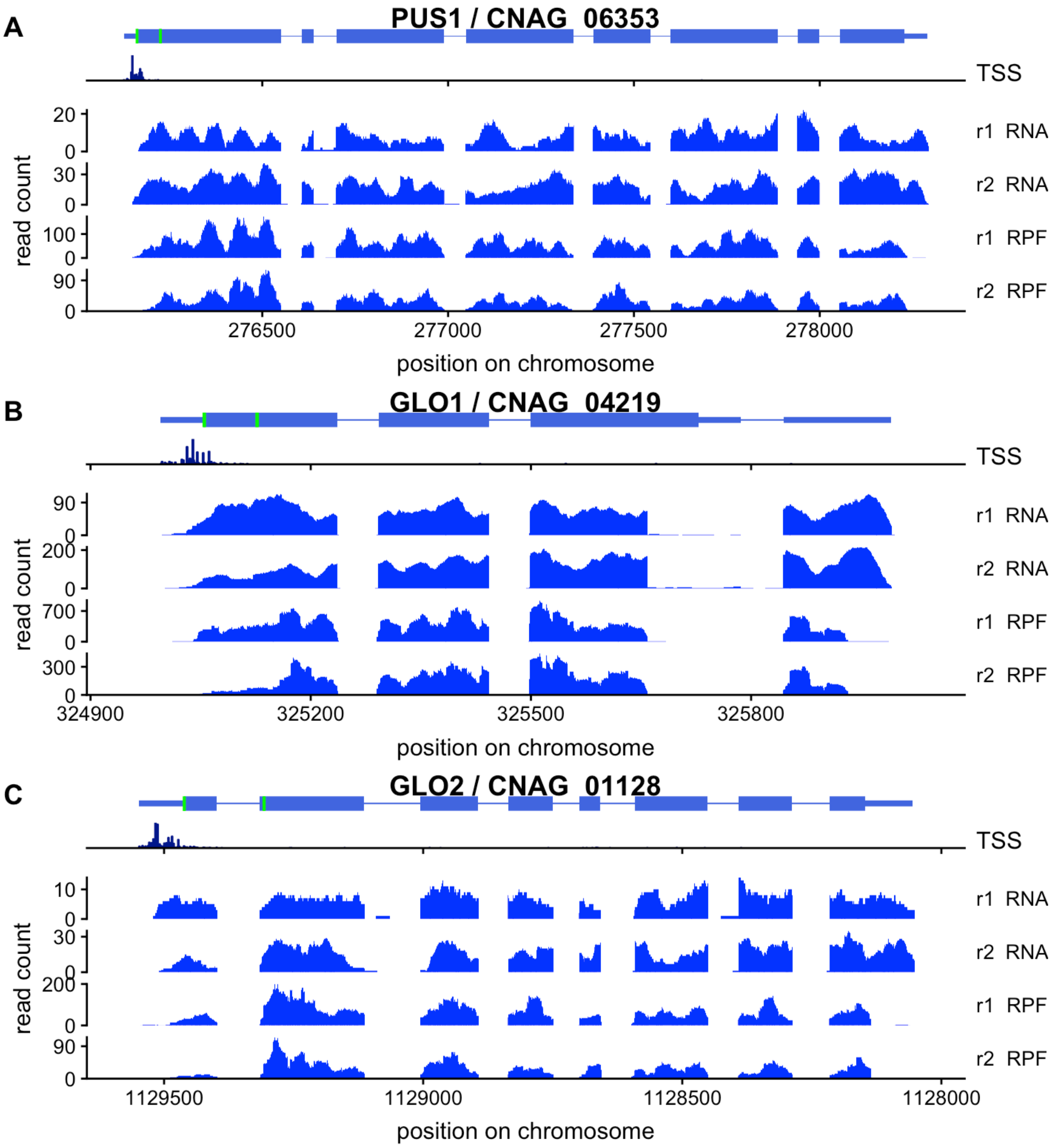

Figure S8: Ribosome profiles of some *C. neoformans* genes with predicted dual-localization specified by alternative N-termini.

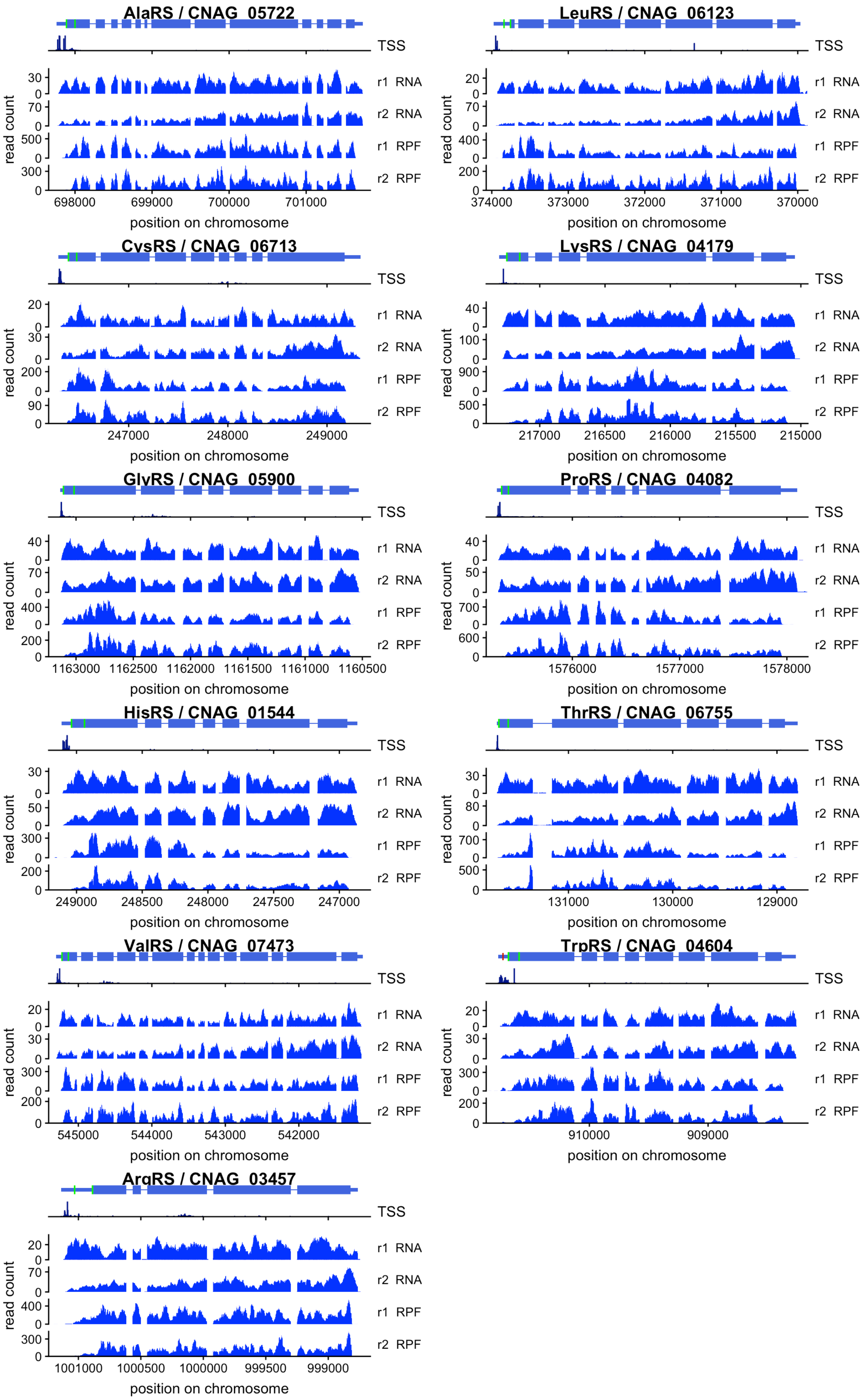
AlaRS / CNAG_05722
LeuRS / CNAG_06123
CysRS / CNAG_06713
LysRS / CNAG_04179
GlyRS / CNAG_05900
ProRS / CNAG_04082
HisRS / CNAG_01544
ThrRS / CNAG_06755
ValRS / CNAG_07473
TrpRS / CNAG_04604
ArgRS / CNAG_03457
TSS
r1 RNA
r2 RNA
r1 RPF
r2 RPF
read count
position on chromosome

Figure S9: Ribosome profiles along the 11 *C. neoformans* aaRS genes with predicted dual-localization. Predicted start codons are shown in green, and the uORF of TrpRS in dark red.

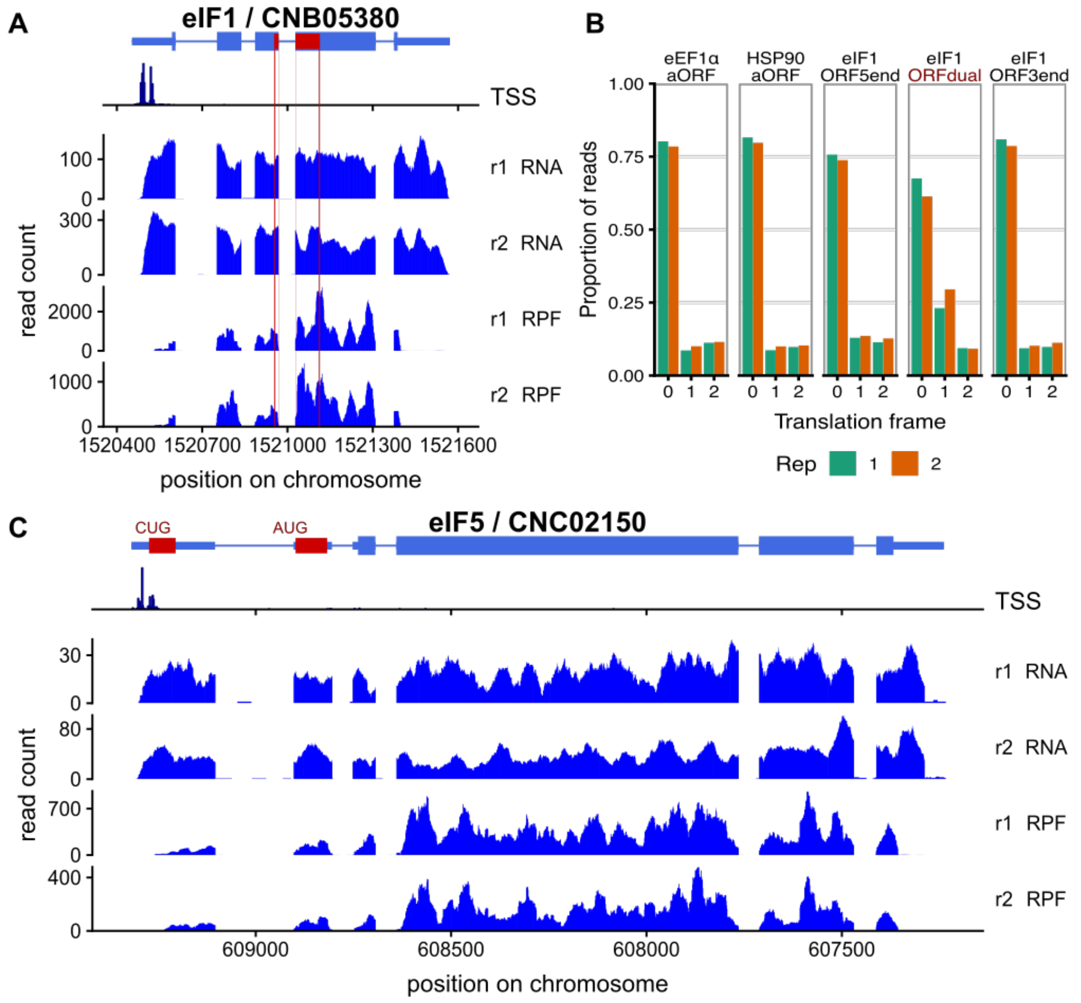

Figure S10: Translation initiation factors eIF1 and eIF5 are regulated by alternate start codon usage in *C. deneoformans*. See figure 8 for legend.

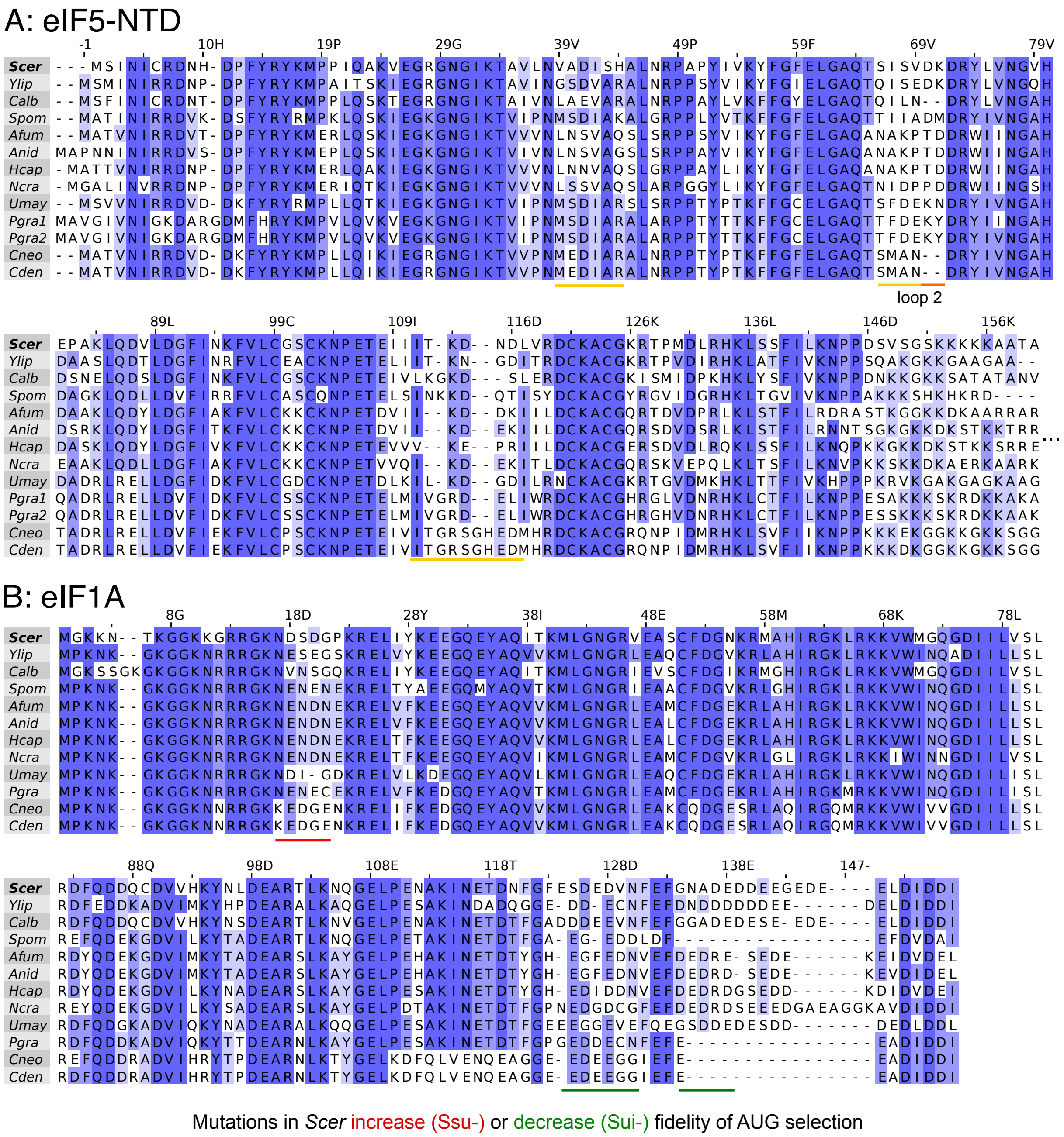

Figure S11: multiple sequence alignments of eIF5-NTD and eIF1A from select fungi. Sequences are numbered according to the *S. cerevisiae* homologs.

Table S1: Sequencing and annotation numbers.

Table S2: Differential expression results in *Cryptococcus deneoformans upf1Δ*.

Table S3: Cytoplasmic ribosomal proteins in 6 fungal species.

Table S4: Genes with score d1AUG – aAUG > 0.1, n = 167.

Table S5: List of aaRS in Cryptococcus and select fungi.

Table S6: Initiation contexts of annotated and downstream AUGs in 9 *Cryptococcus* aaRSs

Table S7: Initiation factor 3 components in 12 fungal species.

Table S8: Oligonucleotides used in ribosome profiling.
